# Wolman’s disease presenting with secondary hemophagocytic lymphohistiocytosis: a case report from Saudi Arabia and literature review

**DOI:** 10.1186/s12887-021-02541-2

**Published:** 2021-02-10

**Authors:** Fahad Alabbas, Ghaleb Elyamany, Talal Alanzi, Tahani Bin Ali, Fatma Albatniji, Huda Alfaraidi

**Affiliations:** 1 Department of Pediatric Hematology/Oncology and Bone Marrow Transplant, Prince Sultan Medical Military Medical City (PSMMC), Sulimaniyah RD, 12233 Riyadh, Saudi Arabia; 2 Department of Central Military Laboratory and Blood Bank, Prince Sultan Medical Military Medical City, Riyadh, Saudi Arabia; 3 Department of Inborn Errors of Metabolism and Genetics, Prince Sultan Medical Military Medical City, Riyadh, Saudi Arabia

**Keywords:** Hemophagocytic lymphohistiocytosis, Lipid storage disorder, Lysosomal acid lipase, Wolman’s disease

## Abstract

**Background:**

Hemophagocytic lymphohistiocytosis (HLH) is a rare and potentially fatal syndrome that is characterized by strong activation of the immune system from hyperinflammatory cytokines. Symptoms of HLH patients include fever, hepatosplenomegaly, cytopenia, and hyperferritinemia. Inherited HLH is classified as primary, whereas secondary HLH (sHLH) occurs when acquired from non-inherited reasons that include severe infection, immune deficiency syndrome, autoimmune disorder, neoplasm, and metabolic disorder. Wolman’s disease (WD) is a rare and fatal infantile metabolic disorder caused by lysosomal acid lipase deficiency, that exhibits similar clinical signs and symptoms as HLH. This paper reports the case of an infant diagnosed with WD and who presented with sHLH.

**Case presentation:**

A 4-month-old infant presenting with hepatosplenomegaly, failure to thrive, and other abnormalities. WD diagnosis was confirmed by the presence of the *LIPA* gene homozygous deletion c.(428 + 1_967-1)_(*1_?)del. The infant also met the HLH-2004 diagnostic criteria.

**Conclusions:**

Metabolic disorder such as WD should be investigated in infants fulfilling the HLH criteria to diagnose the underlying condition. More studies are needed to understand the link between WD and sHLH and to identify appropriate therapies.

## Background

Hemophagocytic lymphohistiocytosis (HLH) is a hyperinflammatory syndrome and one of the most aggressive life-threatening disorders in pediatric hematology. The pathophysiological hallmark of the disorder is a defect in cytotoxic immune function resulting in uncontrolled immune activation, and subsequently systemic hypercytokinemia and widespread organ damage. HLH is characterized by various nonspecific clinical and laboratory abnormalities resembling different disorders. The most common signs and symptoms of HLH are prolonged high fever, hepatosplenomegaly, cytopenia, and laboratory abnormalities including elevated ferritin, triglycerides, transaminases, bilirubin, and lactate dehydrogenase and low fibrinogen [1].

Historically, HLH was classified into two major groups: primary and secondary. Primary HLH is subdivided into two genetically diagnosed sub-groups comprising familial HLH and inherited immune deficiency syndromes. Secondary HLH (sHLH) can be seen in severe infections, rheumatologic disorders, primary immune deficiencies, malignancies, and metabolic disorders. Recently, the North American Consortium for Histiocytosis suggested broader classification of HLH to include other causes of sHLH such as HLH with negative genetic abnormalities and no specific secondary cause, or HLH observed after immune activation (iatrogenic HLH) [2]. As the clinical characteristics of primary HLH cannot be distinguished from sHLH, the diagnosis of HLH is challenging. It is based on molecular diagnosis or presence of five out of eight of HLH-2004 diagnostic criteria which had been created for HLH-1994 trial then modified in HLH-2004 trial [3, 4].

Treatment of HLH is critical and should be started as early as possible to prevent irreversible organ damage. Based on the HLH-2004 trial, the conventional treatment of HLH consists of a potent immunosuppressant (dexamethasone), chemotherapy (etoposide), and standard of care. After remission, the only curative treatment of HLH is allogeneic hematopoietic stem cell transplant (HSCT) which showed improved overall patient survival [5].

Lysosomal acid lipase (LAL) deficiency is a rare, autosomal recessive, and fatal disease characterized by reduced or absent of LAL enzyme activity resulting from mutations in the gene encoding lysosomal acid lipase (*LIPA*). Consequently, the metabolism of cholesteryl esters and triglycerides is impaired. Wolman’s disease (WD) is the infantile form of LAL deficiency [6]. WD patients present with vomiting, diarrhea, increased bilirubin concentration, failure to thrive, hepatosplenomegaly, dyslipidemia, adrenal insufficiency, and hepatic failure. Abdominal X-ray in patients with WD shows bilateral adrenal calcification and the liver biopsy characteristically reveals cholesteryl ester deposits in Kupffer cells [6, 7]. Measurement of LAL enzyme in leucocytes or molecular testing of *LIPA* gene mutations confirms the diagnosis of WD. HSCT and enzyme replacement therapy (ERT) are among the management approaches of WD. Sebelipase alfa is a recombinant human LAL that has been used recently for WD and showed significant improvement in survival and disease symptoms [7].

WD is considered an underlying cause of sHLH where both disorders share common manifestations [8]. Here, we describe the case of a Saudi male infant from first-degree consanguineous parents, who presented with WD and whose condition was subsequently complicated by sHLH. Additionally, alternative cases of WD associated with sHLH that are reported in the literature are reviewed and discussed.

## Case presentation

**Fig. 1:**
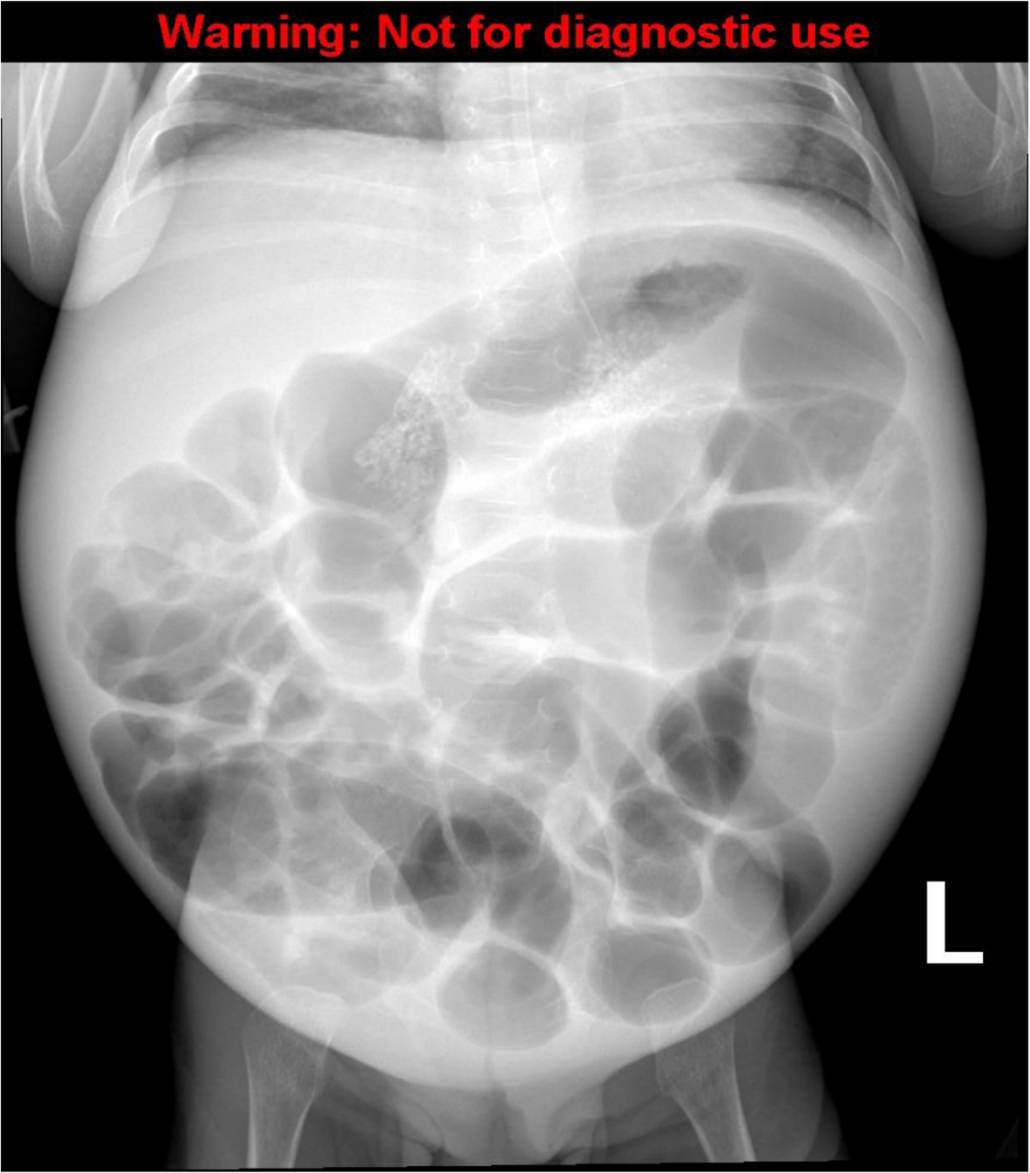
Plain abdominal X-ray showing bilateral adrenal calcification

We report the case of a 4-month-old male infant born full-term after a complicated pregnancy with emergency cesarean section for fetal bradycardia during labor. His Apgar score was 5, 7, and 10 at 1, 5, and 10 minutes, respectively and he stayed 2 days for observation in the neonatal intensive care unit. He is a child of first-degree related Saudi parents. Growth parameters at birth were normal with a weight of 3,250 g, length of 50 cm, and head circumference of 36 cm. At the age of 6 weeks, he needed few days of inpatient care to manage urinary tract infection associated with influenza infection. During an outpatient visit, he was observed to be pale with failure to thrive for an infant of 3 months old. In spite of good oral intake, his weight was 3,700 g, length was 52 cm, and head circumference was 37.5 cm. Based on the child growth standards of the World Health Organization, both weight and length were markedly below the 3rd centile. On physical examination, the infant was active without any apparent dysmorphic features. However, distended abdomen with dilated veins, hepatomegaly (liver span of 8 cm), splenomegaly of 4 cm below costal margin, loss of subcutaneous fat, and reduced muscle bulk were observed. While investigating his failure to thrive, pallor, and organomegaly, the laboratory and radiological work-up showed the following abnormalities (Table 1): severe microcytic anemia, hypothyroidism, elevated serum triglyceride level, hepatosplenomegaly, and osteopenia with bilateral punctuated adrenal calcification which appeared on radiological skeletal survey (Fig. 1).

Cardiac evaluation by echocardiography showed normal heart function. Additionally, while waiting the confirmatory genetic testing, the diagnosis of WD was supported by LAL deficiency where a dried blood spot which showed a very low LAL level.

A deletion/duplication genetic analysis by real-time quantitative PCR (qPCR) confirmed the presence of homozygous deletion c.(428 + 1_967-1)_(*1_?)del in the *LIPA* gene (NM_000235.3; chr.10): (OMIM 613,497). Real-time qPCR amplification (Rotor-Gene Q, QIAGEN, Germany) was done using target gene specific primers. Gene target regions were selected according to the alterations described in the HGMD database, when applicable [HGMD Professional]. The analysis of each fragment was performed in triplicate and by comparison with 4 different normal sample pools. Copy number alterations were calculated according to the Delta Delta Ct method (2^-ΔΔCt), using *LIPA* (NM_000235.3; chr.10): Exons 2, 4 and 10 (or part of the exon) as the reference gene. The detected variant was a homozygous deletion that included, at least, the exons 9 and 10 of the *LIPA* gene. This variant is not described in the literature; however, other deletions have been reported in patients with WD [9, 10]. Based on the available information, this deletion should be classified as a likely pathogenic variant. The enzyme assay was very low which confirms the variant pathogenicity. Genetic testing for the parents was requested to confirm the heterozygous status for genetic counselling.

**Table 1:** Laboratory findings on initial presentation and at time of sHLH diagnosis

Laboratory test	At the diagnosis of WD (3 months old)	At the diagnosis of HLH (4 months old)	Normal reference
WBC	6.9	4.6	5.5–18 × 10^3^/µL
ANC	3.0	3.2	1.0–9.5 × 10^3^/µL
ALC	2.9	1.6	2–17 × 10^3^/µL
Hemoglobin	6.7	6.6	10.1–14.4 g/dL
Platelets count	323	39	150–450 × 10^9^/L
Fibrinogen	NA	1.6	0.9
ALP	153	295	122–469 U/L
ALT	28	229	5–40 U/L
AST	NA	670	5–40 U/L
GGT	NA	222	5–40 U/L
Albumin	34	30	35–55 mg/dL
Total bilirubin Direct bilirubin	7 NA	19 9	0.2–1 mg/dL 0.08–0.25 mg/dL
Serum ferritin	NA	22,683	10–120 ng/mL
LAL activity on leucocytes	< 0.02	< 0.02	0.37–2.30 nmol/punch/hr

*ALC* absolute lymphocyte count, *ALP* alkaline phosphate, *ALT* alanine transaminase, *ANC* absolute neutrophil count, *AST* aspartate transaminase, *GGT* gamma-glutamyl transpeptidase, *HLH* hemophagocytic lymphohistiocytosis, *NA* not applicable, *WBC* white blood cell, *WD* Wolman’s disease

The condition of the infant was managed with supportive care including packed red blood cell transfusion. Although initiation of ERT had been planned,, the medication was not accessible due to limited availability. After the infant condition had been stabilized, he went home on low-fat special formula.

**Fig. 2:**
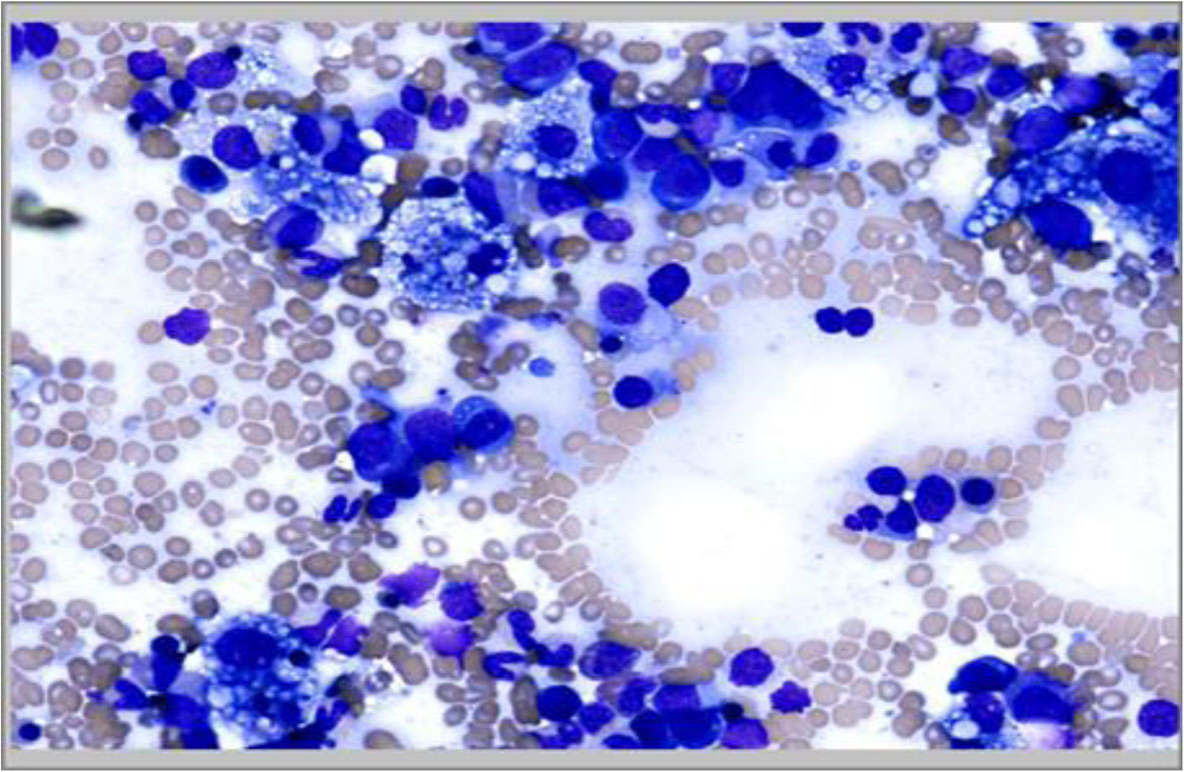
Bone marrow aspirate showing evidence of hemophagocytosis

The infant presented at the age of 4 months with prolonged fever, yellow body discoloration, lethargy, and poor oral intake. Physical examination showed the presence of fever and jaundice in an inactive infant with worsening failure to thrive and hepatosplenomegaly. Laboratory findings revealed pancytopenia, elevated liver enzymes, worsening hypertriglyceridemia, hyperferritinemia, and low fibrinogen level (Table 1). Viral polymerase chain reaction (PCR) for Epstein–Barr virus (EBV), cytomegalovirus (CMV), and adenovirus, and serology for hepatitis B, C, and D were reported to be negative. Blood culture was positive for *Staphylococcus aureus* which was flucloxacillin sensitive. Testing for soluble CD25 level and natural killer cell activity was not available in our institution. In addition to broad-spectrum antibiotics therapy, supportive care including blood transfusion was initiated during the diagnostic work-up. Diagnostic surveillance for immune deficiency and rheumatological disorders revealed absence of hypogammaglobinemia and normal level of anti-nuclear antibodies respectively. Bone marrow aspirate excluded the presence of malignancy and showed the presence of extensive foamy histiocytes with evidence of hemophagocytosis (Fig. 2).

After the infant fulfilled the criteria of HLH, specific treatment according to HLH-2004 protocol was initiated which included dexamethasone 10 mg/m^2^ daily, intravenous immunoglobulin 0.5 g/kg, and cyclosporine 6 mg/kg daily with the exception of etoposide. The infant suffered from venous access difficulty. His condition deteriorated because of refractory thrombocytopenia which required platelets transfusion every few hours to improve platelets count and prevent bleeding during invasive procedure for central line insertion. After the failure of HLH-2004 protocol therapy and because of venous access difficulty, subcutaneous anti-interleukin-1 (anakinra 10 mg/kg twice per day) was initiated. Despite 7 days of the aforementioned treatment, HLH parameters continued to deteriorate in terms of fever, pancytopenia, worsening transaminitis, hyperferritinemia, and hypofibrinogenemia. Additionally, a treatment with 3 doses of etoposide each 150 mg/m^2^ over 10 days instead of anakinra failed to show any improvement. The infant condition progressed to multiorgan failure including liver failure, refractory cytopenia, coagulopathy, and respiratory failure. Unfortunately, the infant died despite the support and intensive therapy provided to manage his condition.

## Discussion and conclusions

Our patient clearly fulfilled the HLH-2004 diagnostic criteria after being diagnosed with WD, based on deficient LAL enzyme. The discovered mutation is novel. Similar to other cases that reported WD and sHLH, the infant showed refractory response to multiple therapies. Including our patient, 11 cases of WD associated with sHLH were reported (Table 2) [11–17]. WD is likely to be underestimated since it is fatal before the likely reorganization of sHLH. Most of WD patients with sHLH are few months old infants and from Middle Eastern communities. Being mostly infants, patients with WD and sHLH might be misdiagnosed with familial HLH without underlying WD, especially that the clinical manifestations are similar in both disorders. Early in the disease, patients with HLH may not meet the full HLH-2004 diagnostic criteria making the diagnosis more difficult [2, 5]. Failure to thrive and huge enlargement of spleen and liver were reported in all patients. Spleen and liver are more enlarged in patients with WD and sHLH compared to familial HLH which might be secondary to the presence of double pathology (lipid storage in addition to excessive immune activation). Failure to thrive, huge organomegaly, and adrenal calcification are likely to be useful in order to suspect underlying WD in infants with HLH.

**Table 2:** Reported cases of patients with WD and sHLH

Study	Country	Presentation	WD diagnosis	Fulfilled HLH criteria	Treatment	Outcome
Al Essa et al. 1998 [11]	Saudi Arabia	4 months with WD and sHLH	Low LAL activity	Fulfilled HLH-1994 criteria	Supportive Specific: NR	Died
Perry et al. 2005 [12]	Canada	2 siblings presented at 49 and 26 days with WD and sHLH	Low LAL activity on autopsy	Presumptive diagnosis	Chemotherapy HSCT	Died
Rabah et al. 2014 [13]	Oman	2 months with WD and sHLH	Low cholesteryl esterase, *LIPA* sequencing negative	Yes	Supportive HLH-2004 protocol	Died
Turasino et al. 2014 [14]	Italy	3 months with WD and sHLH	Low LAL activity	Yes	Supportive Specific: NR	Died
Elsayed et al. 2015* [15]	Egypt	2.5 months with WD and sHLH	Homozygous mutation G969A (W130X)	Yes	NR	NR
Yavus et al. 2017 [16]	Turkey	2 months with WD and sHLH	Low LAL enzyme, heterozygous variation at *LIPA* gene location c:260G > T (GGC > GTC), p.Gly87Val	Yes	Supportive Specific: NR	Died
Tinsa et al. 2019 [17]	Tunis	4 months sHLH and WD	Homozygous mutation c.153 C > A (p.Tyr51*)	Yes	Supportive	Died
Our patient	Saudi Arabia	4 months sHLH in WD	Low LAL activity, homozygous deletion c.(428 + 1_967-1)_(*1_?)del in the *LIPA* gene	Yes	Supportive HLH-2004 protocol	Died

*HLH* hemophagocytic lymphohistiocytosis, *HSCT* hematopoietic stem cell transplant, *LAL* lysosomal acid lipase, *sHLH* secondary hemophagocytic lymphohistiocytosis, *WD* Wolman’s disease, *NR* not reported

*Elsayed et al. reported additional 2 patients with genetically confirmed WD but they did not fulfill HLH criteria

In addition to WD, inborn errors of metabolism such as galactosemia, lysinuric protein intolerance, Gaucher disease, Niemann-Pick disease, methylmalonic acidemia, propionic acidemia, and other disorders have been reported to fulfill the diagnostic criteria of HLH. Whether these disorders should be considered as mimics of HLH disease or have developed immune hyperactivation is unknown. Indeed, the pathophysiology of sHLH in the context of metabolic disorders is not well studied. However, abnormal immune regulation may explain the features of some metabolic disorders [2]. The pathogenic association between the two disorders is not well established as the hyperinflammatory reaction manifested in patients with HLH may be mediated by various triggers. A suggested, trigger of sHLH in metabolic disorders is the induction of inflammation and subsequently macrophage activation by increased metabolites such as cholesteryl ester crystals in WD [14].

Although some reported cases did not describe which specific treatment was provided, the HLH-2004 protocol treatment modality (supportive care with chemotherapy followed by HSCT), has been shown to be associated with inconclusive treatment outcomes and morality rate improvement in WD with sHLH patients. None of the reported studies has used ERT. Due to the advanced patient’s condition, the outcome of ERT was likely to be limited. However, more studies are needed to identify the ability of ERT to prevent HLH complication in WD. In addition to associated significant toxicities, the efficacy of conventional regimens in HLH treatment is limited [18, 19]. More understanding of immune dysfunction in HLH, along with the need for better treatment outcomes, have currently placed targeted therapies under trial. Recently, emapalumab (a monoclonal antibody directed against interferon-gamma) has been shown to be safe and effective in treating progressive, recurrent or refractory primary HLH or cases who are intolerant to conventional treatment [20]. Clinical trials studying the efficacy of emapalumab in treating sHLH are currently ongoing. The success of emapalumab in treating sHLH can be a key factor for managing patients with WD and sHLH.

To conclude, our review confirms the severe or fatal outcome associated with HLH and underlying WD. A High index of suspicion and special precautions are required to identify underlying metabolic conditions such as WD in a patient presenting with HLH. Absence of evidence of familial HLH in infants with HLH should initiate metabolic work up. More studies are needed to understand the link between metabolic disorders and sHLH and to identify the best appropriate treatment for sHLH in patients with WD.

## Data Availability

The datasets generated and/or analysed during the current study are available in the Ensembl genome browser / repository, [https://nov2020.archive.ensembl.org/Homo_sapiens/Share/22fa807b673d90e7f331c8f6fb46618c?redirect=no;mobileredirect=no].

## List of Abbreviations

ALC: Absolute lymphocyte count
ALP: Alkaline phosphate
ALT: Alanine transaminase
ANC: Absolute neutrophil count
AST: Aspartate transaminase
CMV: Cytomegalovirus
EBV: Epstein–Barr virus
ERT: Enzyme replacement therapy
GGT: Gamma-glutamyl transpeptidase
HLH: Hemophagocytic lymphohistiocytosis
HSCT: Hematopoietic stem cell transplant
LAL: Lysosomal acid lipase
NA: Not applicable
NR: Not reported
PCR: Polymerase chain reaction
sHLH: Secondary hemophagocytic lymphohistiocytosis
WBC: White blood cell
WD: Wolman’s disease
